# Niobium-Treated Titanium Implants with Improved Cellular and Molecular Activities at the Tissue–Implant Interface

**DOI:** 10.3390/ma12233861

**Published:** 2019-11-22

**Authors:** Aude Falanga, Pascal Laheurte, Henri Vahabi, Nguyen Tran, Sara Khamseh, Hoda Saeidi, Mohsen Khodadadi, Payam Zarrintaj, Mohammad Reza Saeb, Masoud Mozafari

**Affiliations:** 1 Nancy’s School of Surgery, Universite de Lorraine, F-54011 Nancy, France; audfalanga@gmail.com (A.F.);; 2 Laboratoire LEM3 UMR 7239, Universite de Lorraine, F-57045 Metz, France; 3 Laboratoire Matériaux Optiques, Photoniques et Systèmes, CentraleSupélec, Université Paris-Saclay, F-57070 Metz, France; henri.vahabi@univ-lorraine.fr; 4 Université de Lorraine, CentraleSupélec, LMOPS, F-57000 Metz, France; 5 Department of Nanomaterials and Nanocoatings, Institute for Color Science and Technology, Tehran P.O. Box 16765-654, Iran; 6 School of Chemical Engineering, College of Engineering, University of Tehran, Tehran P.O. Box 14155-6619, Iran; hodaa.saeedi@gmail.com (H.S.); m.kh.599@gmail.com (M.K.); 7 School of Chemical Engineering, Oklahoma State University, 420 Engineering North, Stillwater, OK 74078, USA; payam.zarrintaj@gmail.com; 8 Department of Resin & Additives, Institute for Color Science & Technology, Tehran P.O. Box 16765-654, Iran; mrsaeb2008@gmail.com; 9 Department of Tissue Engineering & Regenerative Medicine, Faculty of Advanced Technologies in Medicine, Iran University of Medical Sciences, Tehran P.O. Box 144961-4535, Iran

**Keywords:** titanium, niobium, biomaterials, implant, interface

## Abstract

There have been several attempts to improve the cellular and molecular interactions at the tissue–implant interface. Here, the biocompatibility of titanium-based implants (e.g., Grade 2 Titanium alloy (Ti-40) and titanium–niobium alloy (Ti-Nb)) has been assessed using different cellular and molecular examinations. Cell culture experiments were performed on three substrates: Ti-40, Ti-Nb, and tissue culture polystyrene as control. Cells number and growth rate were assessed by cell counting in various days and cell morphology was monitored using microscopic observations. The evaluation of cells’ behavior on the surface of the implants paves the way for designing appropriate biomaterials for orthopedic and dental applications. It was observed that the cell growth rate on the control sample was relatively higher than that of the Ti-40 and Ti-Nb samples because of the coarse surface of the titanium-based materials. On the other hand, the final cell population was higher for titanium-based implants; this difference was attributed to the growth pattern, in which cells were not monolayered on the surface. Collagen I was not observed, while collagen III was secreted. Furthermore, interleukin (IL)-6 and vascular endothelial growth factor (VEGF) secretion were enhanced, and IL-8 secretion decreased. Moreover, various types of cells can be utilized with a series of substrates to unfold the cell behavior mechanism and cell–substrate interaction.

## 1. Introduction

A wide range of materials has been utilized in biomedical applications from organic to inorganic materials. Acquainting and acquiring profound knowledge about material properties, composition, and structure results in designing more proper platforms for biomedical applications. For instance, tissue engineering endeavors to architect platforms that mimic the tissue structures and properties [1,2,3,4]. Implants should exhibit appropriate biocompatibility, corrosion resistance, and antibacterial features to be used in the human body [5,6,7,8]. Selecting suitable substrates can be performed by evaluating cells’ performance in contact with suitable substrates, which results in cell adhesion, proliferation, growth, and differentiation [9,10,11,12]. In vitro studies facilitate the prediction of material performance within in vivo applications [13,14,15]. Bioactive glass/gelatin nanocomposite scaffolds coated with bone morphogenetic protein-7 delivered through bone marrow mesenchymal stem cells were used in order to accelerate the regeneration process of bone tissue. It was observed that the scaffold exhibited the osteoinduction effect [16].

It was reported that titanium alloy grade V was functionalized with human collagen for promoting the fibroblast adhesion, which resulted in ameliorating the tissue–implant interactions [17,18]. Epidermal keratinocytes and dermal fibroblasts were cultured simultaneously on nanostructured titanium. The appropriate adhesion of fibroblasts to the titanium surface and proper cell proliferation and differentiation was observed; however, keratinocytes exhibited inappropriate adhesion and proliferation individually. Fibroblasts, in noncontact co-culture with keratinocyte, exhibited proper orientation, proliferation, and enhanced gene expression onto a titanium substrate, while declined adhesion and proliferation was the predominant behavior for the keratinocytes. However, keratinocyte adhered to the surface and proliferated appropriately under contact co-culture condition. It was supposed that the contact co-culturing results in enhancing fibroblasts’ maturation and forming the dermal matrix because of collagen I secretion and growth factor-β1 transformation; these phenomena can promote the proliferation of keratinocytes and create a base membrane via fibroblast inducement to secrete keratinocyte growth factor (KGF), nidogen, and collagen IVα-1 [19].

Jian et al. fabricated a microgrooved plate coated with fibronectin to evaluate the human gingival fibroblasts adhesion, proliferation, and alignment. It was noticed that fibroblasts tend to adhere, proliferate, and align onto coated surfaces. These substrates can be potentially used as dental implants [20]. Ghaffari et al. have used bone marrow-derived human mesenchymal stem (BM-hMSCs) for evaluating the orthopedic and dental titanium implants coated with crystalline apatite. It was used to enhance the osseointegration through increasing implant–bone interface interactions [21]. Wang et al. studied the influence of smooth and rough titanium surface topographies on the macrophage polarization, which affects fibroblast behavior. It was observed that on acid-etched surfaces, macrophages polarize toward a pro-inflammatory (M1) phenotype, which adversely affects the gingival fibroblast behavior [22]. The Ti-40 and Ti Nb were widely used in biomedical applications; hence, the evaluation of interactions between these substrates and cells can provide researchers with valuable information that is useful in implants design. The aim of the present research is to examine the interactions of Ti-40 and Ti Nb surfaces with cells and interpret the cells’ behavior on these substrates.

Titanium alloys TA6V are commonly used in a range of biomedical applications [23]. Recently, beta titanium–niobium (β-Ti-Nb), thanks to its strong mechanical properties (e.g., shape memory effect, superelasticity, and low elastic modulus) and non-cytotoxic constructing elements, has been extensively studied for biomedical applications [24,25,26]. Binary β-Ti-Nb alloys have been recently proposed as an alternative to TA6V due to the biocompatibility of both constituents originating from the ability of both metals to form protective surface oxides [27,28]. However, several critical properties needed for biomedical applications have not been systematically explored for Ti-Nb alloys when compared with TA6V alloy or commercially pure titanium (CP-Ti). These properties include cytocompatibility, corrosion resistance, and altering the surface composition of alloys after prolonged exposure to physiological fluids. Many studies were designed to evaluate the cytocompatibility of Ti-Nb as a way of investigating its corrosion resistance. According to potentiodynamic tests, the observed increased cytocompatibility of Ti-Nb alloys was attributed to reduced ion release and enhanced corrosion resistance as well [29,30]. It has been reported that Ti-Nb alloys possess good corrosion resistance. For clinical applications, cytotoxicity and compatibility with osseous tissue should to be evaluated, while to our knowledge, there is no published study regarding these issues. Accordingly, this study aims to evaluate the cytotoxicity and biocompatibility of the Ti26Nb (at%) alloy, which may be considered as the first step toward its real use in biomedical applications.

## 2. Material and Methods

### 2.1. Materials

Hexamethyldisilane (HMDS), glutaraldehyde, fibroblast Growth Medium (Promocell), Supplement mix (Prom006Fcell), and PrestoBlue (Interchim) were purchased from Merck (Heidelberg, Germany). Trypsin, bovine serum albumin (BSA) in phosphate-buffered saline (PBS), fetal calf serum (FCS), and diluent hemoglobin (HB) were purchased from Sigma-Aldrich (Carol, France).

### 2.2. Methods

#### 2.2.1. Preparation of Ti–26Nb

**Figure 1:**
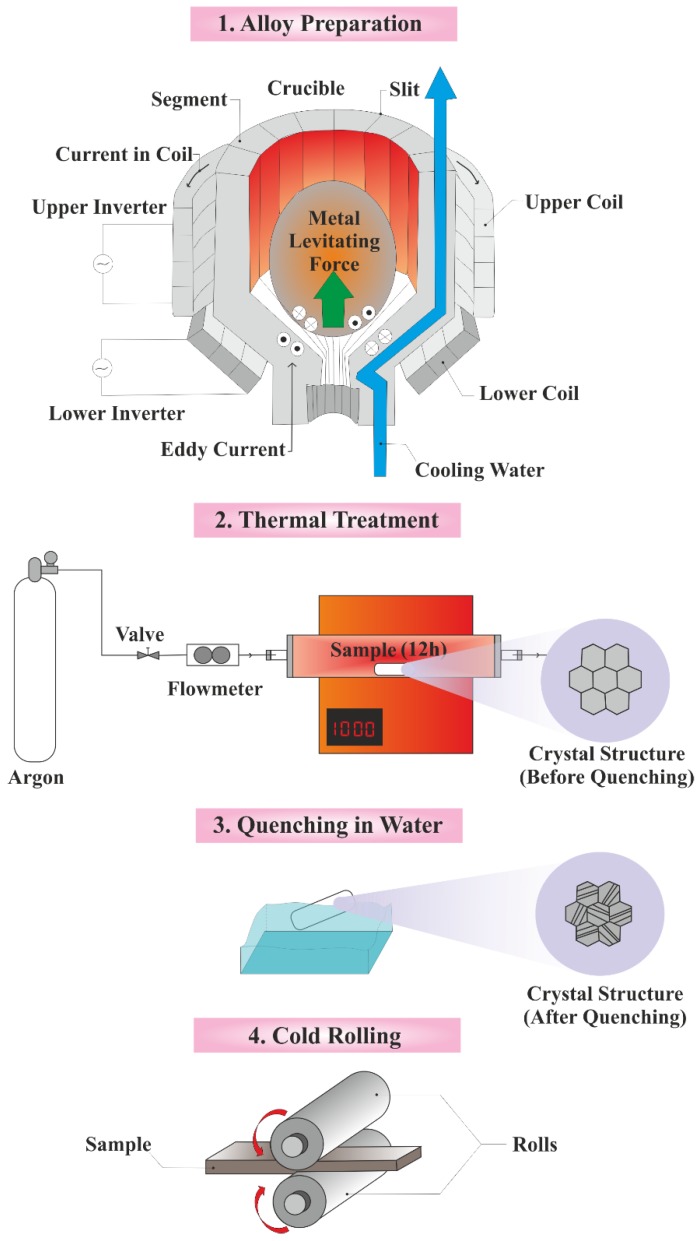
Step-by-step procedure for preparation of Ni- treated Ti: (1) The cold crucible levitation melting technique (CCLM) through which an alloy with a uniform composition is formed by an electromagnetic force. Using this method, metals with a high melting point are melted with no contamination, (2) Tube furnace in which the obtained alloy is homogenized at high temperature under high-purity argon atmosphere, (3) The specimens are quenched to achieve the favorable crystal phase, and (4) Cold rolling with the aim of achieving the favorable sample thickness (source: authors).

The Ti–26Nb (at%) alloys were prepared using the cold crucible levitation melting technique (CCLM) in an argon atmosphere [31]. The elemental analysis of the alloys is presented in Table 1. The obtained ingots were subsequently homogenized at 1223 K for 12 h under an inert argon atmosphere followed by cold rolling until the thickness reached 1.90 mm (true deformation). For the thermal treatment process, the specimens were encapsulated in quartz tubes under a partial pressure of high-purity argon. After breaking the quartz tubes, the resulted specimens were quenched in water in order to obtain the β phase. The samples were cut into discs (diameter: 10 mm and thickness 2 mm); then, the discs were polished mechanically using silicon carbide papers followed by a final polishing step using colloidal silica suspension. The schematic illustration of this procedure is represented in Figure 1.

#### 2.2.2. Contact Angle Measurement and Surface Evaluation

Atomic force microscopy (AFM) was performed using a Park Scientific Instrument (PC) to evaluate surface roughness of the samples. Contact angle measurements were done by a contact angle measuring system model OCA 15 plus as well.

#### 2.2.3. Cells Culture Procedure

The fibroblasts provided by PromoCell (Lot #2080713) and used in this study were extracted from a human donor (57-year-old Caucasian female). The cells were taken from the skin of the temple in May 2011 and frozen at the second pass (Cryo-SFM, PromoCell).

After inoculation, the cells were placed in the incubator under normoxic conditions (37 °C and 5% CO_2_). The culture medium was completely exchanged every 48 h. The medium used for the fibroblasts was “Fibroblast Growth Medium” (Promocell) with the addition of “Supplement mix” (Promocell) and 10,000 U/mL of penicillin and 10 mg/mL of streptomycin.

#### 2.2.4. Growth Monitoring

A 10× dilution of PrestoBlue (Interchim) was carried out (Montluçon, France) using fresh culture medium, and contacted with cells for 2 h at 37 °C in dark. After incubation, 100 µL of the supernatant from each well was taken and placed in a 96-well plate, providing a triplicate for the blank. Then, the well plates were characterized by fluorescence-based spectrometric assay at an excitation wavelength of 560 nm and emission of 590 nm.

#### 2.2.5. Cells Monitoring by SEM

The cells were fixed using 2.5% glutaraldehyde in 0.1 M of cacodylate buffer, pH 7.2, for 1–2 h, at +4 °C. Then, they were rinsed twice using sodium cacodylate buffer. Then, the sample was dehydrated using a scheduled ethanol immersion program, i.e., at 30 °C: 5 min, 50 °C: 5 min, 70 °C: 5 min, 80 °C: 5 min, 90 °C: 5 min, and 100 °C: 20 min. Then, it was dehydrated chemically using HMDS for 10 min. After complete drying, the sample was metallized exposing to a gold/palladium sputtering target for 100 s. The prepared sample was characterized using a Cambridge Stereoscan S 240 Scanning Electron Microscope (SEM, Cambridge, England) at 20 kV of accelerating voltage.

#### 2.2.6. Phenotyping of Fibroblastic Cells

The fibroblasts provided by PromoCell (Lot # 2080713) used in this study were from a human donor (57-year-old Caucasian female), which taken from the skin of the temple and frozen at the second pass (Cryo-SFM, PromoCell). The osteoblasts provided by PromoCell (Lot #3032702) were from a human donor (64-year-old Caucasian male). These are osteoblasts derived from the spongy bone of the femoral head. They were collected and frozen at the second pass in Serum-free medium for cryopreservation (Cryo-SFM). Cells were removed from liquid nitrogen (−196 °C) and thawed in a water bath at 37 °C for 2 min. After enumeration, cells were seeded at 3000 cells/cm^2^ and 30,000 cells/cm^2^ for osteoblasts. After inoculation, the cells were placed in the incubator under normoxic conditions (37 °C and 5% CO_2_). The change of complete culture medium was done every 48 h. The medium used for the osteoblasts was “Osteoblast Growth Medium” (Promocell) with the addition of “Supplement mix” (Promocell), 10,000 U/mL of penicillin, and 10 mg/mL of streptomycin

The cells were first trypsinized (trypsin 0.04% gibco). After separation and counting, they were poured in 1% of bovine serum albumin (BSA) in phosphate-buffered saline (PBS). The labeling was carried out by adding 10 µL of antibody per 100 µL of cell suspension, in which the markers are CD34, CD105, CD90, CD44, and CD45, coupled with a fluorochrome fluorescein isothiocyanate (FITC) or Podocalyxin (PE) or Allophycocyanin (APC) (BD Biosciences). Incubation was performed at room temperature and in the dark for 20 min. Then, the tubes were washed by adding 3 mL of PBS followed by centrifuging at 2500 rpm for 5 min. Then, the supernatant was expelled, and the cells were fixed with a solution of 400 µL 0.5% PBS-formaldehyde.

At first, the standard was reconstituted with 781 µL sample diluent HB + 0.5% BSA and a standard range was achieved through cascade dilutions with a 1:4 ratio. The 96-well filter plate was moistened with 100 µL of assay buffer. Then, the samples were diluted to 750 µg of prot/mL for cytokines and 250 µg of prot/mL for matrix metalloproteinases (MMPs), using sample diluent hemoglobin (HB) + 0.5% BSA. The bead solutions were made, and 50 µL of bead solution was added to each well. Two wash–buffer washes were performed, and 50 µL of each sample was deposited per well. The plate was covered with an adhesive and incubated for 1 h while shaking (850 ± 50 rpm) at ambient temperature followed by washing 3 times. The detection antibody solutions were manufactured using the following procedure: 25 µL of antibody was added per well, and then the plate was incubated at room temperature and in the dark for 30 min while shaking (850 ± 50 rpm). Then, the samples were washed 3 times, and the plate was incubated at room temperature in the absence of light for 10 min while shaking (850 ± 50 rpm). The obtained beads were washed three times and resuspended by adding 125 µL of assay buffer to each well while shaking for 30 s at 850 ± 50 rpm.

#### 2.2.7. Collagen Detection

In a 24-well plate, the cells were saturated with 500 µL of 1% FCS (diluted in PBS) for 1 h. After removing the stromal vascular fraction (SVF), 500 µL of the anti-collagen I and III antibodies was added in PBS/SVF 1% while maintaining at 200 °C for 1 h and 37 °C for 1 h followed by rinsing, three times, through immersion in TBST (Mixture of 1 µL of tris-buffered saline (TBS) and 500 µL of Tween for) for 5 min and expelling the effluents out. Red fluorescence (using a Leica MacroFluo™ Microscope, Nanterre, France) at 499–519 nm (wavelengths range) and 553–568 nm was used for detection of anti-collagen III and anti-collagen I, respectively. At first, the standard solution was reconstituted with 781 µL sample diluent HB + 0.5% BSA, and a standard range was achieved through cascade dilutions with a 1:4 ratio. The 96-well filter plate was moistened with 100 µL of assay buffer. Then, the samples were diluted to 750 µg of prot/mL for cytokines and 250 µg of prot/mL for MMPs with sample diluent HB + 0.5% BSA. The bead solutions were made, and 50 µL of bead solution was added to each well. Washing using wash buffer was performed twice, and 50 µL of each sample was deposited per well. The plate was sealed using an adhesive and incubated at room temperature for 1 h while shaking (850 ± 50 rpm) followed by washing three times.

Image analysis was performed by the imaging platform located at Biopôle. All the results obtained are in arbitrary units and have been normalized with respect to the size of the image, thanks to the Java-based Image J software (National Institutes of Health, Bethesda, MD, USA).

#### 2.2.8. Statistical Analysis

To interpret collected data, Origin Lab pro and MestReNova software (Santiago de Compostela, Spain) were utilized. While the *p* value < 0.05 was considered statistically significant, all the experimental data was analyzed using Student’s *t* test and revealed as mean ± standard derivation (three samples each time). The error bar represents a 68% confidence interval ± s (deviation standard).

## 3. Result and Discussion

**Figure 2:**
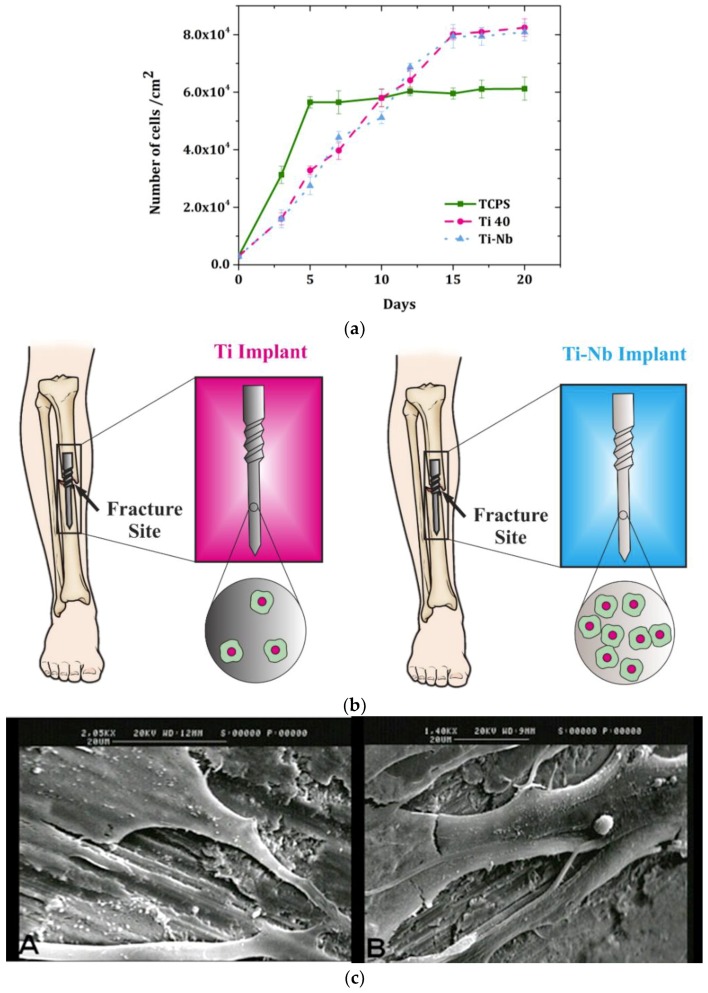
(**a**) Cell viability of fibroblasts under three culture conditions: culture on plastic control sample, Grade 2 Titanium alloy (Ti40), and titanium–niobium alloy (Ti-Nb), (**b**) Ti-Nb implant exhibits proper cell proliferation compared to the Ti implant, (**c**) SEM images of (A) cells grown on Ti40, G = 2050×; (B) cells grown on Ti-Nb, G = 1400×; image indicates the cell adhesion to the surface.

The biocompatibility of Ti40 and Ti-Nb alloys were assessed through culturing the dermal fibroblast (DF) on these substrates. Tissue culture polystyrene (TCPS) plates were used in all experiments as control samples. As shown in Figure 2, the fibroblasts proliferation obeys the exponential behavior in which the cells number reached around 6 × 10^4^ cm^−2^ and 8 × 10^4^ cm^−2^ for TCPS and titanium-based platforms, respectively. Furthermore, the population-doubling time (PDT) for fibroblast cells was around 23, 58, and 57 h for TCPS, Ti40, and Ti-Nb, respectively. In the initial stages, because of the sparse population of fibroblast cells and enough space, cells proliferated rapidly (Figure 2); however, after a while, when cells are densely populated and there was not enough space, the cells proliferation rate declined. The cell proliferation rate was lower for titanium-based platforms because of lower cell adhesion due to their robust structure. However, the initial adherent cell lines secrete an extracellular matrix (ECM) and collagen on the substrate, which makes the surface more appropriate for other cells to attach onto. Consequently, the final population of fibroblast cells on a titanium-based platform is higher that on TCPS. The cell density (cell number/cm^2^) on TCPS reached as high as 5 × 10^4^ with a PDT of 45 h, which is lower than that of fibroblast cells on titanium-based substrates, despite the numerous numbers of initial cells. After 15 days, the difference between samples and TCPS was statistically significant.

Fibroblasts adhesion on titanium-based substrates was evaluated by SEM. As illustrated in Figure 2, fibroblasts did not proliferate in monolayer such that high confluency can be reached for fibroblasts. The fibroblast phenotype was also evaluated as presented in Table 2. The marker had been assessed initially, and after that, at 21 days, the phenotypes of cells were assessed again when cultured on substrates. The results show that the markers’ CD105, CD90, and CD44 values are positive; however, CD34 and CD45 are negative. CD44 is a cell surface, single-pass transmembrane proteoglycan that is expressed in most cell types, which act as a hyaluronic acid (HA) receptor and play a role in cell adhesion and contraction. HA as a CD44 ligand interacts with osteopontin, collagen, and laminin. Moreover, CD44 mediates the fibroblast cells’ behavior. CD44 enhanced proliferation and initial attachment, but declined the strength of cell attachment in high cell density, which is in accordance with Figure 2a. CD90 is known as a positive regulator of osteoblast differentiation and activation; hence, it enhances bone formation while simultaneously inhibiting adipogenesis and obesity. It is noteworthy that the CD105 value after 21 days became negative for all samples. The loss of the marker CD105 could be attributed to differentiation into adipocytes (cells constituting adipose tissue). Different factors may be responsible for the differentiation, such as for example growth hormones, cyclic adenosine monophosphate (cAMP), or glucocorticoids.

**Figure 3:**
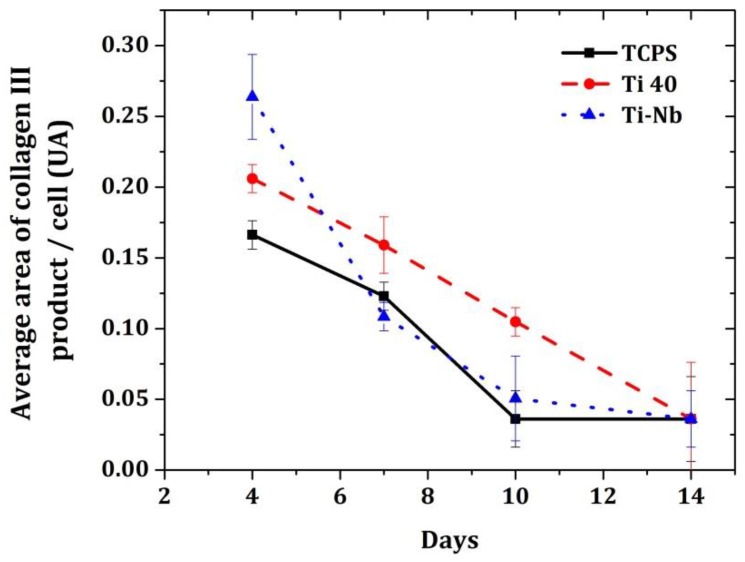
Production of type III collagen by fibroblasts under three culture conditions: culture on TCPS, Ti40, and TiNb.

The average surface area of collagen was measured at day 4, 7, 10, and 14. It was noticed that there is only the collagen type III on these days (Figure 3).

**Figure 4:**
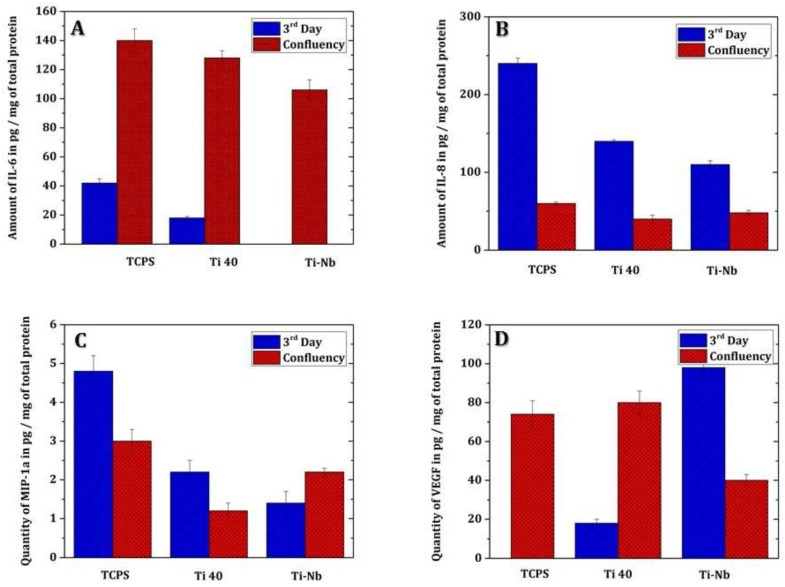
(**A**) Assay of IL-6 at Bioplex, (**B**) Assay of IL-8 at Bioplex, (**C**) Assay of MIP-1a at Bioplex, (**D**) VEGF assay at Bioplex. The data significantly are significant.

The absence of type I collagen could be explained by the fact that during the healing process, type III collagen, which contributes to tissue elasticity, is first synthesized and then replaced by type I collagen, which is responsible for preserving the tissue’s shape. The IL-6 assay was performed on the third day and at confluency (i.e., seventh day for fibroblasts grown on TCPS and 14th day for those grown on Ti-40 and Ti-Nb), as shown in Figure 4A. It should be noted that IL-1β, IL-10, and TNF-α were not detected. Regarding the IL-6 assay, the general pattern was an increase in the amount of IL-6. Indeed, for fibroblasts grown on TCPS, the amount of secreted IL-6 increased from 45.9 µg/mg protein ± 7.3 to 140 µg/mg protein ± 12.3. For fibroblasts cultured on Ti-40, the amount of secreted IL-6 increased from 18.36 µg/mg protein to 129.8 µg/mg protein ± 0.8. Finally, for fibroblasts cultured on TiNb, the amount of IL-6 increased from 0 µg/mg protein to 108 µg/mg protein ± 11 (Figure 4A). Regarding the IL-8 assay, the general pattern was a decrease in the amount of IL-8. For fibroblasts cultured on TCPS, the amount was increased from 235.4 µg/mg protein ± 25 to 66.5 µg/mg protein ± 12. For fibroblasts grown on Ti-40, the amount was increased from 137.8 µg mg/kg protein ± 38 to 38 µg/mg protein ± 14, and for fibroblasts grown on Ti-Nb, the amount was increased from 111 µg/mg protein ± 6 to 47 µg/mg protein ± 29 (Figure 4B). The macrophage inflammatory proteins (MIP-1α) assay showed a relatively stable secretion by fibroblasts over time. The amount secreted by fibroblasts grown on TCPS increased from 5 µg/mg protein ± 3 to 3 µg/mg protein. The amount secreted by fibroblasts grown on Ti-40 had increased from 2.32 µg/mg protein ± 0.4 to 1.28 µg/mg protein. Furthermore, the amount secreted by fibroblasts grown on Ti-Nb had increased from 1.45 µg/mg protein to 2.20 µg/mg protein ± 0.25 (Figure 4C). The VEGF assay showed an increase in the secretion of it by the fibroblasts. The amount secreted by fibroblasts grown on TCPS had increased from 0 µg/mg protein to 72 µg/mg protein. The amount secreted by fibroblasts grown on Ti-40 had increased from 15.15 µg/mg protein to 80.2 µg/mg protein ± 12. Finally, the amount secreted by fibroblasts grown on Ti-Nb had increased from 39.25 µg/mg protein ± 5 at 98.5 µg/mg protein (Figure 4D).

The Bioplex^®^ fibroblast-secreted cytokine assay showed an increase in IL-6 from the time the cells were confluent, a confluent IL-8 decrease, a stable MIP-α over time, and an increase in VEGF at the confluence of cells. IL-6 and IL-8 are considered crucial pro-inflammatory factors in the initiation of periodontitis. IL-6 is responsible for modulation of the inflammation cascade associated with chronic periodontitis. IL-8 is the major chemokine involved in the infiltration of neutrophils into periodontal lesions, inducing the inflammation of periodontal tissues. MIP-1α is a chemokine inducing the migration of monocytes and T cells at the site of inflammation. In vitro, VEGF has the power to induce angiogenesis—that is, the growth of new vessels (neovascularization) from preexisting vessels. VEGF is also a survival factor; it will prevent apoptosis induced by a lack of serum in the culture medium. The profile induced by the titanium plots highlighted by this cytokine assay would be a rather non-inflammatory profile. In fact, apart from the increase in IL-6, which is pro-inflammatory, the decrease in IL-8 and the increase in VEGF are more positive for gingival remodeling. This profile is confirmed by the decrease in the amount of MMP-1, which involves collagenases produced by many cells including fibroblasts and which degrade collagen by enzymatic action.

**Figure 5:**
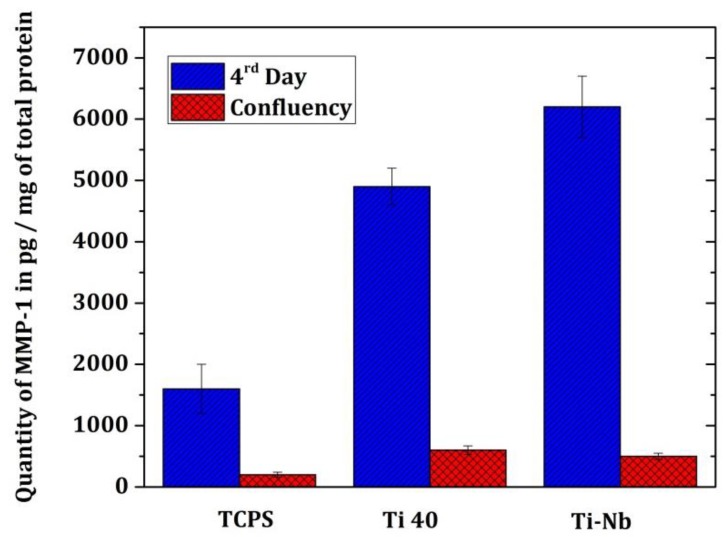
Assay of matrix metalloproteinase (MMP)-1 at Bioplex. The data statistically are significant.

The MMPs were assayed on the fourth day and at confluence (seventh day for cultures on TCPS and 14th day for cultures on Ti-40 and Ti-Nb). Only MMP-1 is assessed, MMPs 8 and 13 have not been detected. The MMP-1 assay showed a decrease in the amount of these. The amount of MMP-1 present in TCPS fibroblast cultures had increased from 1665.2 µg/mg protein ± 725 to 284.3 µg/mg protein ± 161. The amount of MMP-1 present in fibroblasts grown on Ti-40 had increased from 4841 µg/mg protein ± 1837 to 565.36 µg/mg protein ± 217. Finally, the amount of MMP-1 present in fibroblast cultures grown on Ti-Nb had increased from 6358 µg/mg ± 2052 protein to 485.15 µg/mg ± 130 protein (Figure 5).

**Figure 6:**
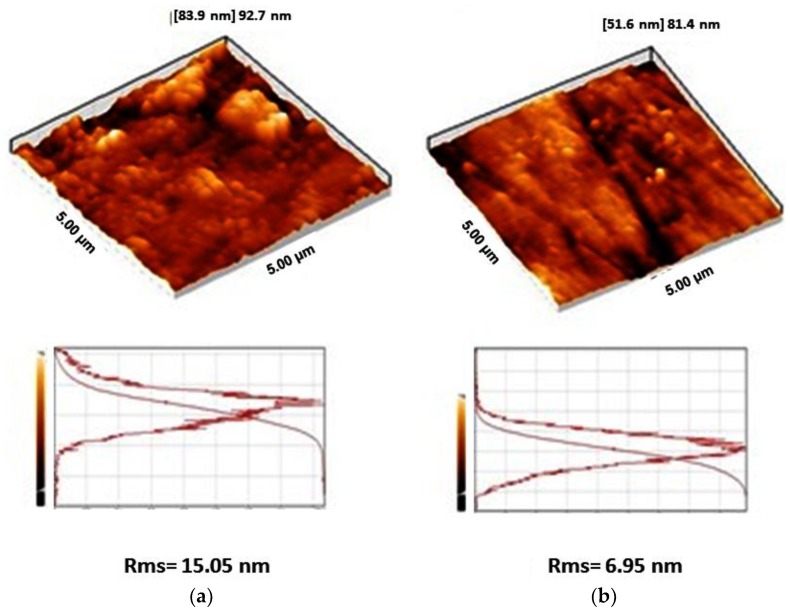
Atomic force microscopy (AFM) images, grain size distribution, and root mean square (rms) roughness values of (**A**) Ti-40 and (**B**) Ti-Nb samples.

The surface morphology and surface roughness of Ti-40 and Ti-Nb samples are compared based on AFM images (Figure 6). AFM images of the samples show a lower grain size for the Ti-Nb alloy (~30 nm) compared to the Ti-40 alloy (about 60 nm). It has been shown that the addition of elements during the formation of an alloy suppresses its grain growth via the formation of thermally stable second phases at the grain boundaries of the host metal phase [32,33]. Moreover, the quantitative analysis of surface roughness of the samples due to AFM images shows a lower root mean square (rms) roughness of Ti-Nb compared to that of Ti-40. It has been previously proved that in aluminum and stainless steel alloys, a larger grain size results in higher roughness values [34,35].

**Figure 7:**
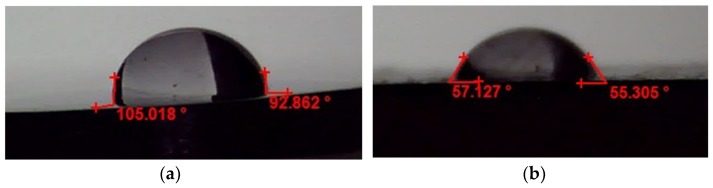
Contact angle values of the samples (**A**) Ti-40, (**B**) Ti-Nb.

The contact angle values of the samples are shown in Figure 7. It can be seen that there is a big difference in the contact angle value of the samples in which the contact angle of Ti-40 is almost twofold that of Ti-Nb. This difference matches well with the rms roughness of the samples (Figure 6). The Ti-40 sample with higher rms roughness is more hydrophobic [36].

Such modification necessitates the equipment for coating, for which the size of the implant should be correlated with the apparatus size. This problem is a limitation of this method. In future studies, the implants will be applied to the animal to evaluate their performance in vivo.

## 4. Conclusions

This study has made it possible to improve the biocompatibility and cytotoxicity of medical implants at the interface. For this purpose, the cellular effects of Ti-40 and Ti-Nb samples have been examined through cell viability, phenotype, collagen secretion, as well as the quantity of integrins, cytokines, and MMPs over time. The surface topology and water contact angle of Ti alloys exhibited different but meaningful values. The Ti-40 contact angle was about 100° and the Ti-Nb was about 56° which it had a proper accordance with roughness values (Rms_Ti-40_ = 15.05 nm and Rms_Ti-Nb_ = 6.95 nm). The titanium plots induced a non-inflammatory profile, but a certain retardation of fibroblast growth. The phenotypic modification of the fibroblasts was not induced by the titanium implants, since it has been also observed on that of control samples during the development. Such treatments can be utilized for controlled cellular activity based on final applications.

## Figures and Tables

**Table 1:** Elemental analysis of alloys.

Elements (wt%)	Nb, Max	N, Max	O, Max	C, Max	H, Max	Fe, Max
Ticp (grade 2)-ASTM	-	0.03	0.25	0.08	0.015	0.2
Ti-Nb	40.5	0.06	0.1	-	0.01	-

**Table 2:** Phenotyping of fibroblasts grown on different substrates. TCPS: tissue culture polystyrene.

Samples	CD105	CD34	CD90	CD44	CD45
Day 0	+78.7%	−0.55%	+100%	+100%	−0.5%
TCPS	−14.16%	−0.2%	+99.8%	+96.63%	−0.63%
Ti-40	−15.47%	0.56%	+99.59%	+98.28%	−36%
Ti-Nb	−13.09%	−0.69%	+99.47%	+94.01%	−0.49%
